# Nanomaterials for Catalysis and Energy Storage

**DOI:** 10.3390/nano13020360

**Published:** 2023-01-16

**Authors:** Sajid Ali Ansari, Nazish Parveen, Md. Mahbubur Rahman

**Affiliations:** 1 Department of Physics, College of Science, King Faisal University, Al Ahsa, P.O. Box 400, Hofuf 31982, Saudi Arabia; 2 Department of Chemistry, College of Science, King Faisal University, Al Ahsa, P.O. Box 380, Hofuf 31982, Saudi Arabia; 3 Department of Applied Chemistry, Konkuk University, Chungju 27478, Republic of Korea

The development of nanomaterials with different shapes and sizes and which are utilized as effective materials for energy and environmental applications constitutes a challenge for researchers [1,2,3,4,5]. This is because our society totally depends on electronic devices, which are certainly made up of and based on various types of energy-storage devices [1,2,3,4,5,6,7,8,9,10]. These devices are assembled with various types of the materials, including metal oxide, hydroxide, metal composites with carbon-based materials such as graphene, conducting polymers, and carbon nanotubes. However, the performance of these materials is sometime not sufficient to completely accommodate the requirements of the daily use of assembled electronic devices. Therefore, various modifications were introduced to improve the overall performance of the materials, such as cyclic stability, energy-delivering performance, durability, and reduction of the fabrication cost of the devices [9,10,11,12,13]. Therefore, this Special Issue focused on the fabrication of the nanomaterials by different methods and examined the associated applications, such as electrochemical performance, catalysis, and energy-storage applications. This Special Issue was assembled with two review articles and eight research articles in different fields of energy storage and catalytic performance of various materials [10,11,13]. Among all the articles, the few articles focused on the importance of the next-generation energy resources serving as alternatives to fossil fuel consumption are increasing every day and, therefore, alternative options should be discussed and explored to avoid future problems [11]. For this purpose, suitable large-scale energy systems should be developed, such as supercapacitors and batteries, owing to their distinguishable characteristics [12]. Therefore, an efficient and suitable current collector which can be used as an energy-storage electrode in various supercapacitors and batteries could be determined and categorized in terms of its stability and cost. For example, three-dimensional nickel foam can be used as an effective current collector in various energy-storage devices [10,11].

This Special Issue also collected various research articles addressing catalysis, which is used in different applications; for example, Boyu Li, et al. [8] reported the stability performance of a ceria-based catalyst. The poor performance of the ceria catalyst in purification industries especially used in diesel particulate filtration due to structural changes occurred during the soot oxidation of the diesel particulate. They controlled the oxygen vacancies and oxygen-storage capacity of the ceria particles by doping the samarium. Bashirul Haq et al. [7] focused on oil recovery using different carbon particles as catalysts derived from date leaves, which are considered to constitute a green method to prepare efficient catalysts. The carbon nanoparticles were prepared from date leaves, which are inexpensive biomass, through pyrolysis and ball-milling methods. The synthesized carbon nanomaterials were characterized using a standard process. Three formulations of functionalized and non-functionalized date-leaf carbon nanoparticle (DLCNP) solutions were chosen for core floods based on phase behavior and interfacial tension (IFT) properties to examine their potential for use in smart water and green chemical flooding. This Special Issues also focused on the advanced articles, such as those addressing the pseudocapacitive behavior of multi-walled carbon nanotubes [4], easy dimeter tuning of silicon nanowire [5], hexagonal-shaped importance in solar cell applications [6], and the importance of the phase changes in thermal-energy storage systems [9]. Overall, this Special Issue will be enriching to interested readers because the collection of articles represents a novel paradigm for energy storage and catalysis. This Special Issue addresses a prevalent engineering problem and provides a novel solution that can become a reference for future studies.

## Data Availability

The data presented in this study are available on request from the corresponding author.
